# Coronavirus disease and home recovery: a Singapore perspective

**DOI:** 10.5365/wpsar.2023.14.5.1003

**Published:** 2023-09-30

**Authors:** Hwee Yong Trevor Tan, Joachim Wen Kien Yau, Matthias Paul Han Sim Toh, Shawn Vasoo, Yee Sin Leo

**Affiliations:** a National Centre for Infectious Diseases, Singapore.; b Headquarters Medical Corps, Singapore Armed Forces, Ministry of Defence, Singapore.; c National Public Health and Epidemiology Unit, National Centre for Infectious Diseases, Singapore.; d Saw Swee Hock School of Public Health, National University of Singapore, Singapore.; e Infectious Disease Research Laboratory, National Centre for Infectious Diseases, Singapore.; f Department of Infectious Diseases, Tan Tock Seng Hospital, Singapore.; g Lee Kong Chian School of Medicine, Nanyang Technological University, Singapore.; h Yong Loo Lin School of Medicine, National University of Singapore, Singapore.

## Abstract

**Objective:**

At the beginning of the coronavirus disease (COVID-19) pandemic in Singapore, the strategy initially involved aggressive ring-fencing of infections, before pivoting towards managing recurrent local interspersed outbreaks of COVID-19. A key feature of Singapore’s efforts to preserve health-care capacity was the implementation of the nationwide Home Recovery Programme (HRP), whereby patients were allowed to recover at home as long as they met certain criteria. The programme was centrally coordinated by Singapore’s Ministry of Health and was supported by telemedicine providers, primary care physicians and government agencies. This report aims to highlight Singapore’s experience in coordinating and implementing the HRP, the challenges faced and the outcomes.

**Methods:**

Published and internal data from the Ministry of Health in Singapore, along with policy documents, were reviewed together with a brief literature review of similar programmes conducted globally.

**Results:**

Implementation of the HRP led to the majority of patients (98%) recovering from COVID-19 in the outpatient setting, with similar mortality rates to inpatient settings. Hospitalization rates for COVID-19 cases were reduced as compared to previously, alleviating strain on the health-care system.

**Discussion:**

The HRP was largely successful at preventing health-care capacities from being overwhelmed, while keeping fatalities to a minimum. Nonetheless, the risks of emergent variants of concern remain present, and heightened vigilance and potential modification of existing protocols based on fluctuations in virulence and infectivity are still needed.

As of 14 February 2023, there have been more than 756 million confirmed cases of coronavirus disease (COVID-19) and 6.84 million deaths related to COVID-19 across 200 countries and territories. (*1*) Singapore was one of the first countries where COVID-19 was detected. (*2*) As of 8 February 2023, the proportion of Singapore residents who had been officially diagnosed with COVID-19 was 37.5% (*n* = 2 220 534), (*3*) although the actual proportion of infections from seroprevalence studies is estimated to be 60%. (*4*) With 1722 fatalities, (*3*) Singapore’s mortality rate of 0.07% was one of the lowest in the world.

During the initial phase of the pandemic, Singapore’s Ministry of Health (health ministry) adopted a policy of compulsory hospital admission and quarantine of all suspected and confirmed COVID-19 cases. As the pandemic progressed, Singapore transitioned towards “living with endemic COVID-19.” As part of this policy shift, home-based recovery became the default for low-risk COVID-19 patients, and a national Home Recovery Programme (HRP), which sought to ensure sufficient health-care capacity at all levels while minimizing morbidity and mortality rates, was implemented. In this brief field investigation report, we summarize the concept of the HRP, the development of its risk-stratification algorithms, experience in implementation, outcomes and future challenges.

## Methods

A PubMed and Ovid MEDLINE search was performed with the terms “COVID-19,” “Pandemic,” “Home Recovery” and “Singapore” for peer-reviewed English-language articles published between 20 January 2020 and 2 March 2023. We also reviewed internal data and policy documents from health ministry, along with information from other government sources and media releases.

## Results

### Home Recovery Programme

Prior to 10 October 2021, all COVID-19 cases in Singapore were triaged into three tiers of facility-based dispositions: community isolation facilities (CIFs), COVID-19 treatment facilities (CTFs) and hospitals. CIFs were facilities for COVID-19 patients who only had mild symptoms but could not isolate at home due to non-medical reasons, while CTFs were facilities with health-care and nursing support that were designed to monitor patients with chronic illnesses who were at risk of deterioration but remained clinically stable. Therefore, low-risk individuals were isolated at CIFs, intermediate-risk individuals were cared for at CTFs, and hospitals were largely reserved for high-risk or acutely unwell individuals. The triaging system implemented was contextualized to suit Singapore’s local needs, to ensure optimal patient placement and delivery of care, while being cognizant of Singapore’s high population density and high basal bed-occupancy rates for non-COVID-19 cases in hospitals.

**Fig. 1:**
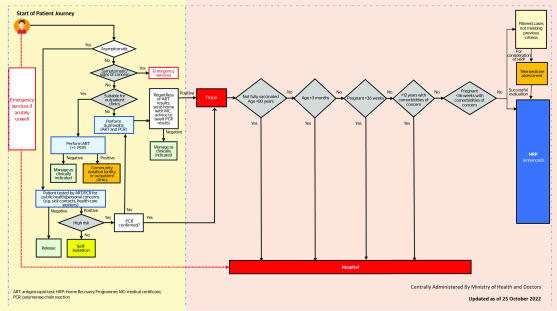
National sorting logic for COVID-19 Home Recovery Programme, 25 October 2022

Beginning on 10 October 2021, a policy transition towards home-based recovery was implemented across two main phases spanning the Delta (from October 2021 to January 2022) and Omicron (from January to June 2022) waves in Singapore. In the first phase, health ministry instituted and administered a national triaging and patient care programme, the HRP, by engaging telemedicine providers and employing technology to remotely triage all newly diagnosed COVID-19 cases, thereby reducing or obviating the need for face-to-face clinical assessments. The HRP triaging system was centrally administered using a hybrid model combining teleconsultation with automation. The centrepiece of this triage was the National Sorting Logic (NSL), which is a stepwise risk-stratification algorithm that serves to determine the initial disposition of each COVID-19 case (**Fig. 1)**. The NSL was developed and periodically updated after analysing local and international data, in consultation with local primary care, infectious disease and public health experts.

[**insert** Figure 1]

Essentially, clinical assessment and triage considered five factors: **C**omorbidities of concern, **A**ge, **V**accination status, **E**xamination/Clinical findings and **S**ymptoms (CAVES). Aided by data integration across national databases, individuals were automatically screened according to their age and vaccination status at the time of diagnosis. High-risk individuals were excluded from the HRP, while remaining patients were further triaged via an online questionnaire that identified potentially vulnerable patients based on comorbidities such as severe immunosuppression and chronic diseases (**Table 1)**.

The questionnaire was highly simplified, requiring only “yes/no” responses, and was translated into all four official languages of Singapore (English, Mandarin, Malay and Tamil). Individuals who answered “no” to all questions were classified as “very low” risk and were enrolled and cared for under the HRP, while individuals with any “yes” responses underwent additional clinical assessment by a telemedicine provider or doctor to assess suitability for the HRP. Similar risk stratification criteria have been used in other countries such as the United States of America (*5*) and the United Kingdom of Great Britain and Northern Ireland. (*6*)

In their care of patients, telemedicine providers operated from a centralized online Telemedicine Allocation and Reconciliation System that served as the COVID-19 case management system and centralized medical records repository for all COVID-19 teleconsultations. (*7*) This consolidated approach allowed for seamless access to data and the tracking of the HRP’s outcomes by health ministry. (*8*) As more data were collected, the NSL was further revised to allow a greater proportion of patients to be safely enrolled in the HRP without needing physical clinical assessment, preserving medical resources without compromising safety.

With the HRP laying the foundation and in anticipation of a larger Omicron wave, health ministry further expanded home-based recovery by empowering primary care physicians to care for individuals in the community whether at low or intermediate/high risk for severe COVID-19, if the patients were clinically stable. Primary care physicians were still able to refer intermediate or high-risk patients for telemedicine based on clinical discretion. National clinical and therapeutic guidelines (*9*, *10*) were developed and disseminated to the Singapore health-care community. Of note, primary care physicians were then able and encouraged to prescribe antiviral treatments, such as nirmatrelvir/ritonavir and molnupiravir, to eligible high-risk patients. (*11*) Besides primary care, oral antiviral treatments were also made available at emergency departments, nursing homes, CIFs and acute hospitals, and their availability facilitated community recovery. This preserved the limited telemedicine and facility-based resources to focus on the intermediate- to high-risk group and prevented hospitals from being overwhelmed.

Concurrently, effective public health messaging and awareness campaigns were also conducted, encouraging patients to recuperate at home and adhere to self-isolation and testing protocols. These included using a combination of mainstream media (television, radio and print) and social media (Instagram, Facebook, WhatsApp and Telegram) for mass outreach. Of note, health ministry set up, maintained and publicized only one official COVID-19 web site (https://www.moh.gov.sg/covid-19) to serve as a single point of reference for the public to obtain up-to-date information on all COVID-19-related health-care policies and key information on what to do when diagnosed with COVID-19. Following a whole-of-government approach, duplicate web sites were avoided to minimize conflicting information and facilitate accurate and timely information dissemination, especially during policy transitions.

### Vaccination

Key to home-based recovery was robust vaccination uptake, with 92% of Singaporeans completing their primary series (defined as two mRNA or Novavax-Nuvaxovid doses, or three Sinovac-CoronaVac doses) and 82% of Singaporeans with minimum protection (defined as three mRNA or Novavax-Nuvaxovid doses, or four Sinovac-CoronaVac doses) as of January 2023. (*3*) With the initial lack of consensus surrounding the optimal number of vaccine doses for adequate protection, especially for the elderly with reduced humoral and cellular immunity, a local study confirmed that a third vaccine dose served to improve immune responses such as memory B-cell and T-cell responses towards the wild type ancestral strains and its variants Delta and Omicron. (*12*) This helped shape the definition of minimum protection being three mRNA doses as defined previously. Data from another study informed that an additional booster with a live-attenuated vaccine such as Sinovac-CoronaVac was required to achieve protection in patients with an initial vaccine primary series of two mRNA vaccinations, which is also correspondingly reflected in the need for an additional booster for those patients receiving Sinovac-CoronaVac. (*13*) Local population data confirmed that most vaccinated patients experienced only mild symptoms and could recover with minimal medical support. (*14*) This was further substantiated by evidence of the milder clinical manifestation of Omicron and its various subvariants relative to previous variants despite their increased transmissibiity. (*15*) An additional mRNA booster, to complete a regimen of four mRNA doses, had also been shown locally to be effective in reducing the risk of hospitalization and severe disease among the elderly aged ≥ 80. (*16*) This provided further impetus for the HRP along with continued public messaging encouraging the elderly to keep their vaccinations up to date.

### Outcomes

Through consistent public messaging, vaccination promotion and leveraging technology, health ministry was able to gradually ease public behaviour and mindsets, preserve national health-care capacities, ensure evidence-based policy adjustments, and maintain low mortality rates throughout the implementation of the HRP. More than 93% of all cases managed to fully recover at home under the programme during the Delta wave (October to December 2021). This proportion increased to 98% during the Omicron wave (January to June 2022), with more patients fully recovering at home under the HRP and primary care. (*17*) During the period when the HRP was implemented (October 2021 to June 2022), there were 1318 deaths out of 1 617 535 cases, for an overall mortality rate of 0.08%.

## Discussion

### Initial challenges and experience in implementation

The implementation of the HRP encountered several challenges, including the definition of comorbidities of concern in the screening checklist, (*17*) along with operational issues in coordination during the surge in COVID-19 cases during the initial roll-out period. (*18*) To accurately describe “comorbidities of concern” in the screening questionnaire, the list of comorbidities that health ministry’s Expert Committee on COVID-19 vaccinations had earlier defined was referenced, while also formulating distinct risk categories for certain conditions. For instance, an initial pragmatic body mass index threshold of ≥ 35 for obesity was set, taking into consideration the weight distribution in Singapore. However, subsequently a more straightforward cut-off point of 100 kg was selected for the sake of simpler execution. As for operational issues encountered during the implementation of the HRP, additional agencies such as the Singapore Armed Forces and the People’s Association (a statutory board that promotes social cohesion among neighbourhood committees) were enlisted to increase support for telemedicine and patient communications. (*19*)

### Future challenges

Through effective utilization of technology, mobilization of primary care and government agencies, widespread vaccination, and the deployment of therapeutic countermeasures, Singapore successfully implemented its novel HRP strategy nationwide, preserved health-care capacities and ensured minimum fatalities. It is anticipated that with the maintenance of up-to-date vaccinations and the wider use of oral antivirals in higher-risk patients, the mainstay of COVID-19 care will continue to be largely managed by patients at home or in primary health-care settings. More real-world data will be needed, however, to inform future vaccination strategies for COVID-19 after a primary course or initial boosters are completed, and to define the cost effectiveness and optimal deployment of oral antiviral treatments.

### Conclusion

Regardless of the uncertainty, the lessons learned and strategies developed thus far will likely remain relevant in dealing with future variants or pandemics. Singapore’s experience shows that it is possible to minimize morbidity and mortality in an unprecedented pandemic. It involved a combination of responsive and calibrated public health policies informed by science. The concept of the HRP, enabled by an NSL, should remain adaptive and ready to respond to new variants of concern with differing characteristics and to future pandemics.
